# The Inhibition and Variability of Two Different RT-qPCR Assays Used for Quantifying SARS-CoV-2 RNA in Wastewater

**DOI:** 10.1007/s12560-022-09542-z

**Published:** 2023-02-15

**Authors:** George Scott, Nicholas Evens, Jonathan Porter, David I. Walker

**Affiliations:** 1 Centre for Environment, Fisheries and Aquaculture Science, The Nothe, Barrack Road, Weymouth, DT4 8UB UK; 2 Environment Agency, National Monitoring, Starcross, Exeter, EX6 8FD UK

**Keywords:** SARS-CoV-2, Wastewater, RT-qPCR, Inhibition, Variability, Quantification

## Abstract

Faecal shedding of severe acute respiratory syndrome coronavirus 2 (SARS-CoV-2) and its subsequent detection in wastewater turned the spotlight onto wastewater-based epidemiology (WBE) for monitoring the coronavirus-disease 2019 (COVID-19) pandemic. WBE for SARS-CoV-2 has been deployed in 70 countries, providing insights into disease prevalence, forecasting and the spatiotemporal tracking and emergence of SARS-CoV-2 variants. Wastewater, however, is a complex sample matrix containing numerous reverse transcription quantitative PCR (RT-qPCR) inhibitors whose concentration and diversity are influenced by factors including population size, surrounding industry and agriculture and climate. Such differences in the RT-qPCR inhibitor profile are likely to impact the quality of data produced by WBE and potentially produce erroneous results.

To help determine the possible impact of RT-qPCR assay on data quality, two assays employed by different laboratories within the UK’s SARS-CoV-2 wastewater monitoring programme were assessed in the Cefas laboratory in Weymouth, UK. The assays were based on Fast Virus (FV) and qScript (qS) chemistries using the same primers and probes, but at different concentrations and under different cycling conditions. Bovine serum albumin and MgSO_4_ were also added to the FV assay reaction mixture. Two-hundred and eighty-six samples were analysed, and an external control RNA (EC RNA)-based method was used to measure RT-qPCR inhibition. Compared with qS, FV showed a 40.5% reduction in mean inhibition and a 57.0% reduction in inter-sample inhibition variability. A 4.1-fold increase in SARS-CoV-2 quantification was seen for FV relative to qS; partially due (1.5-fold) to differences in reverse transcription efficiency and the use of a dsDNA standard. Analytical variability was reduced by 51.2% using FV while qS increased the number of SARS-CoV-2 negative samples by 2.6-fold. This study indicates the importance of thorough method optimisation for RT-qPCR-based WBE which should be performed using a selection of samples which are representative of the physiochemical properties of wastewater. Furthermore, RT-qPCR inhibition, analytical variability and reverse transcription efficiency should be key considerations during assay optimisation. A standardised framework for the optimisation and validation of WBE procedures should be formed including concessions for emergency response situations that would allow flexibility in the process to address the difficult balance between the urgency of providing data and the availability of resources.

## Introduction

The coronavirus-disease 2019 (COVID-19) pandemic’s causal agent is severe acute respiratory syndrome coronavirus 2 (SARS-CoV-2). The first reported cases of COVID-19 were linked to markets in Wuhan, China and SARS-CoV-2 has since spread rapidly across the globe (Holmes et al., 2021). As of October 2022, over 629 million cases of COVID-19 had been reported and it has been linked to the deaths of over 6 million people world-wide in just 35 months (Dong et al., 2020). CoVs are enveloped viruses with a + ssRNA genome ranging from 26 to 32 kb. The majority of CoV infections in animals result in gastrointestinal (GI) symptoms with human CoV infections predominantly involving the respiratory system (Su et al., 2016).

The primary transmission route of SARS-CoV-2 in humans is through airborne droplets and aerosols expelled from the lungs of an infected person. Respiratory symptoms and fever were the initial focus for SARS-CoV-2 triage. GI symptoms, however, develop in some clinical cases ca. 3 to 20 days prior to respiratory symptoms (Buscarini et al., 2020; Jones et al., 2020). This indicates that faecal shedding may allow early detection of SARS-CoV-2 in wastewater, although to our knowledge detection of SARS-CoV-2 RNA in faeces prior to respiratory symptom development has not been reported.

Detection of SARS-CoV-2 RNA in human faeces occurs in ca. 54.7% of patients (75 of 137) with shedding in faeces predicted to peak 0.34 d after the onset of respiratory symptoms, continuing for up to 40 days following the onset of symptoms and a week after the last detectable respiratory sample using reverse transcription quantitative PCR (RT-qPCR) (Han et al., 2020; Kim et al., 2020; Liu et al., 2020; Ng, Chan and Chan, 2020; Wang et al., 2020; Wölfel et al., 2020; Zhang et al., 2020; Zhang et al., 2021; Zuo et al., 2021; Miura et al., 2021; Vaselli et al., 2021). Recovery of viable SARS-CoV-2 from faeces, however, is uncommon with only a few studies successfully isolating viable virus from a minority of patients (6 out of 72) (Fei Dergham et al., 2021; Pedersen et al., 2022; Wang et al., 2020; Wölfel et al., 2020; Xiao et al., 2020; Zhang et al., 2020). Following the detection of SARS-CoV-2 in wastewater, there has been an unprecedented global increase in the use of wastewater-based epidemiology (WBE) with 70 countries using WBE to support efforts to control COVID-19 as reported by Naughton et al., (2021) on the COVIDPoops19 dashboard.

WBE has been used for decades as part of the poliovirus eradication programme and has also been used as a tool to monitor other pathogens such as hepatitis viruses and norovirus, antimicrobial resistance, alcohol, tobacco, therapeutic drug use and exposure to chemicals (Asghar et al., 2014; O’Keeffe, 2021; Polo et al., 2020). The technologies and processes required to quantify a viral pathogen in wastewater are simple. Generally, a wastewater sample is collected, the sample is clarified, the viral particle is concentrated, nucleic acids are extracted, and the pathogen is detected or quantified using PCR-based methods. If employed correctly, this can provide the data for downstream use in WBE which has the potential to provide an accurate account of the spatiotemporal changes in the prevalence of human pathogens by allowing a direct quantitative measurement of the causative agent of disease in the served population with fewer anthropogenic biases.

WBE has several benefits over clinical epidemiology including, but not limited to, the detection of asymptomatic cases or cases from persons resistance to self-reporting due to the imposed consequences. This may improve the accuracy of disease prevalence calculations, but such estimates are problematic to validate. For instance, McMahan et al., (2021) used WBE to predicted that levels of SARS-CoV-2 in the community were 11-fold greater than the number of clinical cases which aligned to some degree with other modelling techniques. WBE can also track and quantify variants of concern (VOC) and identify the emergence of new variants without mass testing. Agrawal et al., (2022) reported that the tracking of genomic variants and quantification of VOCs using WBE was consistent with clinical data across 54 European cities. WBE can also be used for forecasting disease as shown by Galani et al. (2020) and Morvan et al. (2022) who identified that changes in wastewater signals appeared 4 to 5 days earlier than in clinical testing and could predicted clinical cases and hospital and ICU admissions 2 to 8 days in advance which could allow pre-emptive control and response measures to be put in place.

Despite the simple concept of WBE, producing high-quality, accurate and reproducible data is challenging and quality issues can at any stage of the process. Sample collection- and processing-related quality issues, however, are not the focus of this study and are reviewed in detail by Polo et al., (2020), Pino et al., (2021) and Ahmed et al., (2022). The challenges of producing high-quality WBE data, however, continue beyond quantification of SARS-CoV-2. Normalisation of SARS-CoV-2 concentrations is required to account for variations in population feeding the sewershed, faecal load and the dilution of domestic wastewater with industrial waste and stormwaters (Hsu et al., 2022; Sweetapple et al., 2022; Wilde et al., 2022). This has been achieved using physical measurements such as flowrate and the quantification of biomarkers such as pharmaceutical and recreational drugs and their metabolites, markers of excretion such ammonia and microorganisms such as cross assembly phage and pepper mild mottle virus (Been et al., 2014; Hsu et al., 2022; Wilde et al., 2022; Wilder et al., 2021). In this study, however, we are concerned with the impact of implementing different RT-qPCR assays on data quality.

One of the most challenging aspects for maintaining quality within a WBE programme is the heterogeneity of wastewater as a sample matrix. The physicochemical characteristics and diversity of RT-qPCR inhibitors in wastewater are largely uncharacterised but are likely to be influenced by several factors. These include the size of the population feeding into the wastewater catchment, the time of collection, turbidity and faecal solids content, site of sampling in the wastewater system, type of wastewater system (combined or separate), the surrounding built and natural environment, climate and the surrounding industries, agriculture and environment. Numerous RT-qPCR inhibitors, however, have been identified in environmental water samples and stool, and are therefore highly likely to be present in wastewater. These include polysaccharides, bile salts, lipid, urate, fulvic and humic acids, metal ions, algae and polyphenols (Schrader et al., 2012; Sims & Kasprzyk-Hordern, 2020).

RT-qPCR inhibitors can disrupt both reverse transcription and qPCR leading to inter-sample differences in reverse transcription and amplification efficiency. Inhibitors have multiple modes of action and can interact with nucleic acids, enzymes or co-factors and prevent the reverse transcriptase or polymerases from progressing along the template or block enzyme activity (Bessetti, 2007). Although not strictly due to RT-qPCR inhibition, contaminants can also quench the fluorescent signals measured during qPCR leading to reduced accuracy (Sidstedt et al., 2020). This aims of this study are, therefore, to determine how the implementation of different RT-qPCR assays within SARS-CoV-2 wastewater monitoring programmes can impact the quality of the data with a specific focus on the impact of RT-qPCR inhibition.

## Materials and Methods

### Sample Collection and Processing

Two-hundred and eighty-six wastewater samples were collected on behalf of the Environment Agency (EA) on 26/10/21 from sites across England managed by 9 different water companies. Approximately 1 L of sample was collected in sterile polyethylene terephthalate (PET) bottles using either a grab or composite method. Samples were then sent by temperature-controlled courier at 5±3 °C to the EA laboratory, Exeter, UK where they were processed on the day of arrival as described by Walker et al., (2022).

Approximately 200 mL of each wastewater sample was clarified by centrifugation at 10000 × g for 30 min and 150 mL of clarified supernatant was mixed with 60 g ammonium sulphate. Samples were incubated for ≥ 1 h at 4 °C and then centrifuged at 10000 × g for 30 min at 4 °C. The supernatant was discarded, and the pellet resuspended in 2 mL of NucliSENS® lysis buffer (BioMérieux, France) in 24-well deep-well KingFisher™ plates (Thermo Fisher Scientific, US). To each of the sample lysates, 50 µL of NucliSENS® magnetic silica beads (BioMérieux, France) were added and the nucleic acids were purified with the NucliSENS® magnetic extraction reagents (BioMérieux, France) using a KingFisher™ Flex (Thermo Fisher Scientific, US) robot fitted with a 24-sample head and 24-well deep-well KingFisher™ plates (Thermo Fisher Scientific, US). The nucleic acid washing procedure was as follows; two washes in 385 µL wash buffer 1, two washes in 485 µL wash buffer 2, one wash in 500 µL wash buffer 3. Nucleic acids were eluted into 120 µL wash buffer 3 and stored at −80 °C prior to being sent to the Cefas laboratory in Weymouth, UK in November 2021. Extracts were sent on dry ice and stored at -80 °C upon receipt. Samples were analysed at Cefas in November 2021 within a single freeze–thaw cycle and stored on ice or at 4 °C when not in use.

### RT-qPCR

Two different RT-qPCR assays were assessed in this study. The first used qScript XLT 1-Step RT-qPCR ToughMix (Quantabio, US) and the second TaqMan™ Fast Virus 1-Step Master Mix (Applied Biosystems, US) according to Farkas et al., (2022) and Kevill et al*.*, (2022); referred to herein as qS and FV, respectively. Both assays used the same primer and probe sequences targeting the SARS-CoV-2 N1 nucleocapsid region producing a 72 bp amplicon (Lu et al., 2020). Total reaction volumes were 20 μl, made up of 15 μl of mastermix and 5 μl of sample. Primers and probes were purchased from MERCK (Germany) and Thermo Fisher Scientific (US) and component concentrations and cycling conditions were as stated in Table 1 and Table 2. The FV assay also had the addition of 50 ng/μl bovine serum albumin and 800 pM MgSO_4_ per-reaction. All samples, standards and controls were run in duplicate with ramping set at 1.6 °C/s using a QuantStudio™ 3 qPCR machine (Thermo Fisher Scientific, US). The background fluorescence threshold limit and Cq values were automatically determined on a plate-to-plate basis using the Design and Analysis Software v2.6 (Thermo Fisher Scientific, US).

**Table 1:** Primer and probe sequences and concentrations for the two assays based on Fast Virus (FV) and qScript (qS) chemistries

Component	Sequence (5′ to 3′)	Concentration (nM)	
		qS	FV
Forward primer	GACCCCAAAATCAGCGAAAT	400	500
Reverse primer	TCTGGTTACTGCCAGTTGAATCTG	400	1000
Probe	FAM-ACCCCGCATTACGTTTGGTGGACC-MGB	100	250

**Table 2:** One-step RT-PCR cycling conditions for the two assays based on Fast Virus (FV) and qScript (qS) chemistries

Cycle stage		Time (s)		Temperature ( °C)	
		qS	FV	qS	FV
Reverse transcription		1200	1800	52.0	55.0
RT inactivation		600	600	96.0	95.0
Denature	× 45	15	15	94.0	95.0
Anneal & extension		60	60	60.0	60.5

Each plate was run with a dsDNA standard curve consisting of a four point, tenfold serial dilution in TEX buffer (Tris–EDTA; 10 and 0.1 mM at pH 8.0 with the addition of Triton X at 0.1% (v/v)) with a starting concentration of 10^5^ copies per μL. dsDNA for the standard curve was produced by the amplification of the N1 nucleocapsid region flanked by 5’ and 3’ primer sites from a synthetic dsDNA plasmid (Thermo Fisher Scientific, US); Table 3. Conventional PCR was performed using the GoTaq® Flexi system (Promega, US) in 50 µL reactions containing 1 × GoTaq® Flexi buffer, 2.5 mM MgCl_2_, 1.225 U Taq, 0.25 mM of each dNTP; primer concentrations and sequences are in Table 3. PCR was performed in an Mastercycler® Nexus thermal cycler (Eppendorf, Germany) using the following conditions: 94 °C for 3 min then 40 cycles of 1 min at 94 °C, 1 min at 55 °C, 1 min at 72 °C and then 72 °C for 7 min.

**Table 3:** Oligonucleotide information and per-reaction concentration for production of the dsDNA standards using PCR.

Component	Sequence (5′ to 3′)	Concentration (nM)
Synthetic oligonucleotide*	GCTATGACCATGATTACGCCAAGACCCCAAAATCAGCGAAATGCACCCCGCATTACGTTTGGTGGACCCTCAGATTCAACTGGCAGTAACCAGATTCACTGGCCGTCGTTTTACA	0.4
Forward primer	GCTATGACCATGATTACGCCAA	1000
Reverse primer	TGTAAAACGACGGCCAGTGAA	1000

*The synthetic oligonucleotide was contained within a synthetic dsDNA plasmid

Amplification was confirmed by agarose gel electrophoresis and the PCR amplicon was purified into 50 µL of elution buffer using the Wizard® SV Gel and PCR Clean-Up System (Promega, US) following the manufacturer’s recommendations. Following purification, the PCR amplicon was quantified with a Qubit™ 2.0 fluorometer (Thermo Fisher Scientific, US) using the Qubit™ dsDNA BR assay kit (Thermo Fisher Scientific, US) according to the manufacturer’s recommendations. The PCR amplicon was diluted to 1×10^5^ copies/μL with TE buffer with 2 ng/µL sheared salmon sperm DNA (Thermo Fisher Scientific, US), split into 50 μL aliquots and stored at −80 °C until required.

### RT-qPCR Inhibition

An external control RNA (EC RNA) method was used to determine RT-qPCR inhibition, whereby the mastermix was spiked with 1 μl of EC RNA per-reaction to a concentration of approximately ~ 200,000 gene copies per μl to minimise the influence of endogenous viral RNA. EC RNA was obtained from a heat inactivated SARS-CoV-2 culture RNA extract kindly supplied by Dr Christine Tait-Burkard of the Roslin Institute, Edinburgh, UK. Eighty-five microlitres of inactivated SARS-CoV-2 was added to 1 mL of NucliSENS® Lysis Buffer and incubated at room temperature for 10 min. The lysate was then loaded into a Maxwell® RSC PureFood GMO and Authentication Kit cartridge (Promega, US) and extracted on a Maxwell® RSC 48 instrument (Promega, US) following the manufacturer’s guidelines and then eluted into 100 μl elution buffer. EC RNA was quantified using the FastVirus RT-qPCR assay and then diluted in TE buffer (Tris–EDTA; 10 and 0.1 mM at pH 8.0). Each of the spiked samples were run in duplicate and an EC RNA-water reference reaction was run with each plate to allow the relative calculation of inhibition.

### Reverse Transcription Efficiency

To determine reverse transcription efficiency, a 111 bp Ultramer® synthetic RNA oligonucleotide (Integrated DNA Technologies, US) with the sequence 5’-GCGGGUCCUGCCGAAAGUAGACCCCAAAAUCAGCGAAAUGAUCACAUUACUGGCCGAAGCCACCCCGCAUUACGUUUGGUGGACCCUCAGAUUCAACUGGCAGUAACCAGA-3’ was used. It was supplied at 10^5^ gc/µL and diluted to 10^4^ gc/µL with TEX. Five independent dilutions were performed and RT-qPCR reactions were run with FV and qS as previously outlined. Each independent dilution was run with two technical repeats and a dsDNA standard curve and negative control as previously outlined.

### Data Analysis

RT-qPCR data were quality checked before proceeding to data analysis. For quantification, standard curves were deemed acceptable if the slope of the log_10_ nucleic acid concentration (gc/µL) vs. C_q_ was between −3.6 and −3.1 with an *R*^2^ of > 0.98. Amplification efficiency was calculated following Eq. 1. For analysis of RT-qPCR inhibition and SARS-CoV-2 quantification, samples with technical repeat ΔC_q_ > 0.5 were excluded from further analysis. For analysis of assay technical repeat variability and detection rates, however, technical repeats with ΔC_q_ > 0.5 were retained but SARS-CoV-2 negative samples were removed for the former.

1 $$AE={D}^{\left(\frac{-1}{S}\right)}$$

Equation 1. Amplification efficiency (AE) where D is the fold dilution used in the standard curve and S is the slope of the standard curve.

RT-qPCR inhibition was calculated using Eq. 2 and for comparative analysis of the two assays’ inhibition levels, samples exhibiting no inhibition were assigned a value of 0. For correlation between the two reagents, negative inhibition levels were not adjusted. SARS-CoV-2 concentration was calculated following Eq. 3 and then log_10_ transformed prior to analysis. Reverse transcription efficiency was calculated following Eq. 4. Technical repeat variability in SARS-CoV-2 concentration was calculated using Eq. 5. Inhibition, quantification and reverse transcription efficiency data from sample duplicates were averaged prior to analysis.

2 $$\text{RT-qPCR inhibition}=\left(1-{10}^{\Delta Cq/m}\right)\times 100\%$$

Equation 2. Calculating RT-qPCR inhibition where ΔCq = Cq value (sample RNA + EC RNA) − Cq value (water + EC RNA) and m = slope of the dsDNA standard curve as defined in ISO 15216–1:2017

3 $$C= {10}^{\frac{Cq-It}{s}}$$

Equation 3. Calculating template concentration (C) in (gc/µL) where Cq is the average Cq for a given sample, It =the intercept of the standard curve for a given RT-qPCR plate and s = the slope.

4 $$RTE\%=100\left(\frac{C}{KC}\right)$$

Equation 4. Reverse transcription efficiency (RTE%) where C = the template concentration as determined using Eq.3and KC is the known concentration of the input material

5 $$TRV=100\left(\frac{{C}_{max}-{C}_{min}}{{C}_{avg}}\right)$$

Equation 5. Normalised technical repeat variability (TRV) where C_min_, C_max_and C_avg_ are the minimum, maximum and average concentration of SARS-CoV-2 for a given sample as calculated in Eq. 3.

For comparative analysis of inhibition and quantification from the two different RT-qPCR assays, only samples with data available for both reagents were used. Paired *t*-tests were performed unless the difference between observations failed to meet the assumption of normality, in which case a Wilcoxon signed-rank test was used. Correlations were calculated using Pearson’s or Spearman’s correlation depending on whether the assumption of normality was violated. For comparison of the sample and reaction detection rates McNemar’s test was performed. Statistical analysis was performed in R and statistical significance is defined at *p* < 0.05.

## Results & Discussion

### Inhibition

**Fig. 1:**
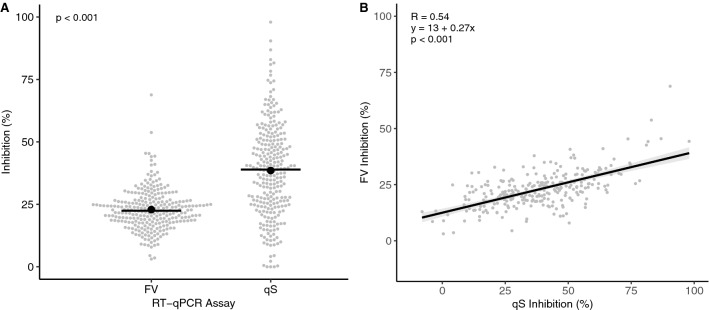
RT-qPCR inhibition in wastewater RNA extracts from two SARS-CoV-2 assays based on Fast Virus (FV) and qScript (qS) chemistries. Data show the distribution of inhibition levels (**A**) and correlation between assays (**B**). Grey markers show the average sample inhibition values. Solid lines and black markers show the group medians and means (**A**) and shaded bars show the 95% confidence interval (**B**); *n* = 266

All RT-qPCR plates run in this study passed the quality control criteria set out previously, and all NTC reactions were negative (Table 4). Two-hundred and sixty-six samples met the quality criteria and were used in the comparative analysis of assay inhibition. For qS, 8 samples (3%) failed to meet the recommended threshold of < 75% inhibition according to ISO standard 15216–1:2017 as used for viral quantification in food, while all FV samples passed (Fig. 1A) (ISO, 2017). Maximum inhibitions were 98.0 and 68.8% for qS and FV respectively, and mean inhibition levels were significantly greater for qS (*p* < 0.001) with a 68.1% increase in mean inhibition from 22.9% for FV to 38.5% for qS (Fig. 1A). Inter-sample inhibition variability was smaller for FV as seen by a reduced standard deviation of 8.1% compared to 18.4% for qS. A moderate positive correlation (*R* = 0.54, *p* < 0.001) was also seen between the qS and FV inhibition levels indicating that samples tended to be challenging for both assays (Fig. 1B).

**Table 4:** Quality parameters for the RT-qPCR assays based on the Fast Virus (FV) and qScript (qS) chemistries used in this study showing±1 standard error of the mean

Quality parameter	FV	qS
R^2^	0.998 ± 0.000	0.999 ± 0.000
Slope (Cq)	−3.304 ± 0.020	−3.407 ± 0.019
Intercept (Cq)	37.578 ± 0.119	37.855 ± 0.146
Amplification efficiency (%)	100.858 ± 0.903	96.66 ± 0.927

The mechanisms causing FV’s reduced susceptibility to inhibition have not been explored and are likely multi-faceted due to numerous differences between the two assay chemistries and cycling conditions. The incorporation of BSA, however, is likely to play a key role and has been shown to bind phenolics such as tannic, humic and fulvic acid present in water and faeces preventing their interaction with and inhibition of reverse transcriptase and polymerases (Giambernardi et al., 1998; Kreader, 1996). Additionally, as inhibitors can interact with enzyme-cofactors, the addition of exogenous of MgSO_4_ in FV will increase the ratio of free- to inhibitor bound-Mg^2+^ and the probability of successful polymerase-cofactor interaction and strand elongation, maintaining amplification efficiency in the presence of inhibitors. Potential differences in polymerase between the two assays, may play a role as inhibition is polymerase-dependent with Taq mutants (N-terminal deletion) showing a 10 to 100-fold increase in resilience to inhibition from blood (Kermekchiev et al., 2009; Matheson et al., 2010). Furthermore, the differences in primer and probe concentrations between the two assays may also contribute towards the difference in sensitivity to inhibitors. Increased primer concentrations as observed for FV may increase the likelihood of a successful reverse transcriptase- or polymerase-primer-template interaction as the proportion of inhibited enzyme increases.

### Quantification, Quality and Detection Rates

**Fig. 2:**
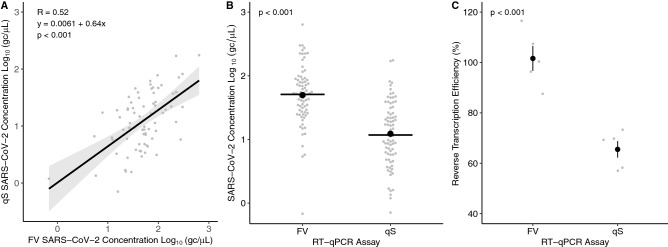
The SARS-CoV-2 concentration from wastewater RNA extracts using two RT-qPCR assays based on the Fast Virus (FV) and qScript (qS) chemistries showing the correlation between quantification (**A**), distribution of SARS-CoV-2 concentrations (**B**) and reverse transcription efficiency (**C**). Grey markers show the average sample quantity. Shaded bars are the 95% confidence interval (**A**) solid lines and black markers show the group medians and means (**B** and **C**); *n *= 77 (**A** and **B**) and 5 (**C**)

Seventy-seven out of 286 samples (27%) gave data suitable for comparative analysis of SARS-CoV-2 quantification. A significant, moderate positive correlation (*R* = 0.52, *p* < 0.001) was seen between the quantification data from qS and FV indicating congruence between the methods (Fig. 2A). Furthermore, a significant increase (*p* < 0.001) in mean SARS-CoV-2 concentration was seen for FV with a 0.61 log_10_ (gc/µL) or 4.1-fold difference between assays (Fig. 2B). These differences may be due to the increased levels of inhibition observed for the qS assay (Fig. 1B) or inefficiencies in reverse transcription (RT). Reverse transcription efficiency was measured by quantification of RNA at known concentrations for FV and qS. A significant 1.5-fold difference (*p* < 0.001) was found in RT efficiency between FV and qS with mean RT efficiencies of 101.1 and 65.5% for FV and qS, respectively (Fig. 2C).

This indicates that the differences in SARS-CoV-2 quantification are not solely due to RT efficiency and that cycling conditions, primer concentration and inhibition are likely to play a role; although the nature of this study did not allow the additional contributing factors to be determined. These data, however, indicate that RT efficiency should be optimised in addition to amplification efficiency to ensure the accuracy of quantification by RT-qPCR, as they can act independently on assay performance. Furthermore, it highlights the importance of using RNA standards for RT-qPCR to allow accurate quantification. The long-term stability of the RNA standards, however, should be monitored to prevent RNA degradation impacting the accuracy of results. Where this isn’t possible, confirming the equivalence RNA and DNA standards may be appropriate. Although this may miss batch-to-batch differences in reverse transcriptase efficiency or the impact of multiple freeze–thaw cycles on performance.

**Fig. 3:**
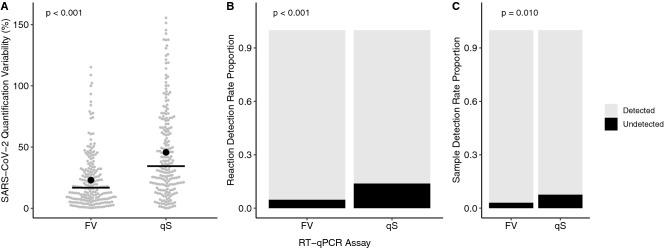
The normalised technical repeat variability in SARS-CoV-2 quantification from two RT-qPCR assays based on the Fast Virus (FV) and qScript (qS) chemistries as calculated by *Eq.*5 (**A**), detection rates for technical repeats (**B**) and samples as determined by a positive result in at least one of the technical repeats (**C**). Solid lines and black markers show the median and mean (**A**); *n *= 205 (**A**), 528 (**B**) and 264 (**C**)

In addition to impacting assay accuracy, the different assays also influenced the precision of quantification with the median normalised variability between technical repeats being significantly reduced (*p* < 0.001) by 51.2% from 34.4 to 16.8% for the FV assay relative to qS (Fig. 3A). Furthermore, the proportion of reactions and samples that tested positive for SARS-CoV-2 increased significantly for FV relative to qS (*p* = 0.009 and < 0.001) from 86.2 and 92.2% to 96.3 and 97.0%, respectively (Fig. 3B & C). This equates to a 2.6-fold increase in the number of undetected samples when using qS, indicating FV may be more sensitive and have the potential to lower the limit of detection (LOD) and quantification (LOQ) of SARS-CoV-2 RNA in wastewater.

Due to the design of the study and proprietary nature of the different assay chemistries, determining the factors contributing to assay variability is difficult. Inhibitors are likely to play a role and the level of variability is likely to be inhibitor- and inhibitor concentration-dependent but also dependent on the diversity of inhibitors present in a sample. Along with primer and probe concentrations, BSA is likely to play a role in reducing variability and the differences in detection rates as it has previously been shown to reduce inhibition, alter sensitivity in an assay-dependant manner and improve detection rates in blood, faeces, meat and food rinsate (Al-Soud and Rådström, 2000; King et al., 2009; Plante et al., 2011). These observations give further focus for assay optimisation procedures and highlight that analytical variability should be considered and most importantly, representative sample matrices should be used during these procedures.

## Conclusions

In this study we observed that two of the RT-qPCR assays being implemented within the SARS-CoV-2 wastewater monitoring programmes in the UK have significantly different accuracies, precisions, detection rates and inhibition levels when they are used to quantify the same target in the same wastewater sample; all of which were processed at the same facility using the same viral concentration procedure. Each of the assays had different mastermix chemistries, the same primer and probe sequences but at different concentrations and had similar cycling parameters. It is important to remember, however, that many WBE initiatives for SARS-CoV-2 were implemented as rapidly as possible under difficult circumstances. The choice of reagents being used by labs was in-part driven by their availability due to issues with the supply chain and reagents being reserved for clinical testing.

Significantly reduced susceptibility to inhibitors (40.5%) and reduced inter-sample variability in inhibition levels (56.0%) were observed for FV relative to qS. These factors will directly impact the accuracy of data and reduce the confidence in any models produced by WBE. These findings highlight the importance of using representative samples and monitoring RT-qPCR inhibition during assay optimisation as minimising inhibitor-induced bias is vital for accurate quantification in all qPCR applications. Furthermore, optimisation to minimise the impact of inhibitors should be performed for all differing assay chemistries and targets independently as inhibition is amplicon-dependent (Huggett et al., 2008).

For WBE, intensive assay performance assessment on a broad range of wastewater samples with varying physicochemical properties and target concentrations should be performed for assay optimisation and validation. Determining acceptable levels of RT-qPCR inhibition for WBE programmes will be dependent on the nature of the data’s use (e.g. qualitative detection or disease forecasting) and will ultimately be a trade-off between accuracy, precision, sensitivity, specificity, cost, reagent availability and the limitations of the laboratories implementing the programme. We recommend that the planning and preparation for any pandemic in which WBE may be applicable should recognise these issues. Furthermore, investments should be made to research the diversity of RT-qPCR inhibitors in wastewater to allow appropriate methods to be developed to mitigate their impact and improve the quality of data produced using WBE.

Following the implementation of inhibition mitigation techniques, it would be best practice to continue monitoring inhibition until greater confidence in the WBE procedures can be determined. This can be achieved using either an internal amplification control (IAC), EC RNA or serial dilutions of the sample with appropriate data correction techniques being applied if validated. Each of the inhibition monitoring techniques has its own advantages and disadvantages and should be chosen on a per-target and application basis as expected copy number, throughput and availability of funds for method development will play a part in the final decision.

IACs rely on of the quantification of a unique oligonucleotide and can be performed as a multiplex reaction in parallel to the quantification of the amplicon of interest. IACs can be competitive or non-competitive using either the same primers but a different probe to the amplicon of interest or separate primers and probe, respectively. The independent nature of IACs allows inhibition to be monitored at environmentally relevant concentrations as the normalisation of endogenous template isn’t required as is for EC RNA. IACs, however, require extensive optimisation to ensure that inhibition levels are consistent between the amplicon of interest and synthetic sequence and that the assays work correctly in multiplex.

EC RNA, as used in this study has the disadvantages of requiring at least one additional reaction per sample and still requires validation to be used for data correction to overcome the assumption that inhibition is equal at all template concentrations. Arguably, the most favourable way to achieve accurate quantification is to ensure that amplification efficiencies are equal between sample and standards using multiple dilutions for each sample. This method, however, is extremely consumable and labour intensive and can lead to issues with quantification in situations such as WBE where low template concentrations are present.

In addition to RT-qPCR inhibition susceptibility, the two assays showed significantly different SARS-CoV-2 concentration with FV exhibiting a 4.1-fold increase in SARS-CoV-2. This was in-part due to differences in RT efficiency which led to a 1.5-fold difference in SARS-CoV-2 concentration; which is likely to have an impact on assay sensitivity, accuracy, LOD and LOQ. The differences in quantification are likely to be, in-part, resolved by using a representative RNA standard. Differences in detection rate, and potential impacts on LOD and LOQ, however, are unlikely to be resolved without further optimisation of reverse transcription and reducing assay sensitivity to inhibitors. Reducing the impact of inhibitors is likely to play an integral role in increasing the accuracy of microbial quantification from wastewater and, thus, the reliability and usefulness of data produced in WBE. As such, comparative analysis of RT-qPCR-based quantification techniques should be performed against other methods which may be more robust to inhibitors such as digital PCR.

Analytical variability was reduced by 51.2% for FV while qS increased the number of SARS-CoV-2 negative samples by 2.6-fold. In combination with the observations of reduced inhibition, increased RT efficiency and increased quantification of SARS-CoV-2 from FV, this study initially highlights the obvious—thorough assay optimisation using representative samples is required to produce high-quality data. This is especially important when the data may be used to guide policy decisions in situations such as a pandemic.

Crucially, this study highlights, however, that we were not fully prepared to respond to an outbreak from a novel pathogen which to a certain extent can be expected from a field of research still in its relative infancy. We should take this opportunity to learn valuable lessons for the future and work towards a standardised approach or at least a formal framework for assay optimisation and the validation of methods for WBE. This should also consider emergency procedures and outline the minimum levels of assay optimisation and validation required for WBE so that assays can be developed quickly as new situations arise. For this to be successful, however, a greater understanding of the physiochemical nature of wastewater is needed on both a local, national and international level so that effective and efficient sample collection procedures, processing methods and quantitative assays can be developed and stress-tested prior to implementation.
